# Effects of higher PEEP and recruitment manoeuvres on mortality in patients with ARDS: a systematic review, meta-analysis, meta-regression and trial sequential analysis of randomized controlled trials

**DOI:** 10.1186/s40635-020-00322-2

**Published:** 2020-12-18

**Authors:** Lorenzo Ball, Ary Serpa Neto, Valeria Trifiletti, Maura Mandelli, Iacopo Firpo, Chiara Robba, Marcelo Gama de Abreu, Marcus J. Schultz, Nicolò Patroniti, Patricia R. M. Rocco, Paolo Pelosi, For the PROVE Network: PROtective Ventilation Network

**Affiliations:** 1 Department of Surgical Sciences and Integrated Diagnostics, University of Genova, Largo Rosanna Benzi 8, 16131 Genova, Italy; 2 Anesthesia and Intensive Care, Ospedale Policlinico San Martino IRCCS per l’Oncologia e le Neuroscienze, Genova, Italy; 3 Department of Intensive Care, Academic Medical Center, University of Amsterdam, Amsterdam, Netherlands; 4 Department of Critical Care Medicine, Hospital Israelita Albert Einstein, São Paulo, Brazil; 5 Pulmonary Engineering Group, Department of Anaesthesiology and Intensive Care Medicine, University Hospital Carl Gustav Carus, Technische Universität Dresden, Dresden, Germany; 6 Mahidol Oxford Tropical Medicine Research Unit (MORU), Mahidol University, Bangkok, Thailand; 7 Nuffield Department of Medicine, University of Oxford, Oxford, UK; 8 Laboratory of Pulmonary Investigation, Carlos Chagas Filho Institute of Biophysics, Federal University of Rio de Janeiro, Rio de Janeiro, Brazil

**Keywords:** Acute respiratory distress syndrome, Positive end-expiratory pressure, Mechanical ventilation

## Abstract

**Purpose:**

In patients with acute respiratory distress syndrome (ARDS), lung recruitment could be maximised with the use of recruitment manoeuvres (RM) or applying a positive end-expiratory pressure (PEEP) higher than what is necessary to maintain minimal adequate oxygenation. We aimed to determine whether ventilation strategies using *higher* PEEP and/or RMs could decrease mortality in patients with ARDS.

**Methods:**

We searched MEDLINE, EMBASE and CENTRAL from 1996 to December 2019, included randomized controlled trials comparing ventilation with *higher PEEP* and/or RMs to strategies with lower PEEP and no RMs in patients with ARDS. We computed pooled estimates with a DerSimonian-Laird mixed-effects model, assessing mortality and incidence of barotrauma, population characteristics, physiologic variables and ventilator settings. We performed a trial sequential analysis (TSA) and a meta-regression.

**Results:**

Excluding two studies that used tidal volume (*V*_T_) reduction as co-intervention, we included 3870 patients from 10 trials using higher PEEP alone (*n* = 3), combined with RMs (*n* = 6) or RMs alone (*n* = 1). We did not observe differences in mortality (relative risk, RR 0.96, 95% confidence interval, CI [0.84–1.09], *p* = 0.50) nor in incidence of barotrauma (RR 1.22, 95% CI [0.93–1.61], *p* = 0.16). In the meta-regression, the PEEP difference between intervention and control group at day 1 and the use of RMs were not associated with increased risk of barotrauma. The TSA reached the required information size for mortality (*n* = 2928), and the z-line surpassed the futility boundary.

**Conclusions:**

At low *V*_T_, the routine use of higher PEEP and/or RMs did not reduce mortality in unselected patients with ARDS.

**Trial registration:**

PROSPERO CRD42017082035.

## Introduction

Despite intense research, mortality of patients with acute respiratory distress syndrome (ARDS) remains high [1]. Respiratory support is mandatory in ARDS to maintain adequate gas exchange, but mechanical ventilation itself can contribute to further lung damage in a process referred to as ventilator-induced lung injury (VILI). The main determinants of VILI are high pressures and volumes and cyclic opening and closing of respiratory units [2]. Development of VILI may translate into an iatrogenic component of ARDS mortality, overlapping with that due to the underlying lung condition, and can be reduced by optimising mechanical ventilation [3].

Ventilation settings aimed to minimise VILI are referred to as ‘protective mechanical ventilation’. However, different putative mechanisms of VILI have been targeted, and there is no unanimous consent on which ventilator settings should be considered ‘protective’ or ‘more protective’. After the encouraging results of the first trials comparing bundles of interventions such as tidal volume (*V*_T_) size reduction, positive end-expiratory pressure (PEEP) and recruitment manoeuvres (RMs) with conventional ventilation [4, 5], new debates have arisen to determine which of these parameters improved outcome. In a trial conducted by the ARDS network, both arms received the lowest PEEP/FIO_2_ combination necessary to achieve an acceptable oxygenation; however, the group receiving *V*_T_ = 6 mL per kg of predicted body weight (PBW) resulted in lower mortality compared to 12 mL/kg [6].

Subsequently, the use of PEEP levels higher than those strictly required to maintain oxygenation (‘*higher’* PEEP) with or without the concomitant use of RMs has been proposed in the so-called ‘open lung approach’ strategy, aimed at maximising lung recruitment during ventilation [7]. Accordingly, the authors proposed different methods to titrate PEEP, either based on oxygenation or respiratory mechanics goals, and trials investigated whether mortality could be further reduced by applying *higher* PEEP on a routine basis. A recent clinical practice guideline of the American Thoracic Society, European Society of Intensive Care Medicine and Society of Critical Care Medicine advocates the use of *higher PEEP* in patients with moderate or severe ARDS [8], based on the results of an individual data meta-analysi s[9]. However, this guideline was published before the latest trials and did not stratify studies according to the use of *higher PEEP*, RMs or their combination.

We conducted a systematic review and meta-analysis of RCTs comparing ventilation strategies comprising *higher PEEP* and/or RMs to conventional strategies with lower PEEP levels and no RMs, either used alone or in combination. We aimed to determine whether the routine use of *higher* PEEP and/or RMs could reduce mortality in ARDS patients. We hypothesized that the indiscriminate use of *higher PEEP* and/or RMs in all ARDS patients had no effect on mortality.

## Methods

### Data sources and searches

This review evaluated randomised trials in patients with ARDS, which investigated ventilation strategies that included *higher PEEP* levels and/or RMs (intervention) versus fixed PEEP or PEEP increased stepwise enough only to reach minimal oxygenation goals (control). The eTable 1 contains details on patients, interventions, comparators and outcomes.

We classified as *‘higher PEEP’* any strategy resulting in or aimed at obtaining PEEP levels higher than those achieved in the control group, in which PEEP was kept at a fixed level or increased enough only to reach minimal adequate oxygenation goals. We considered ‘RM’ any transient increase in airway pressure aimed at restoring or improving lung aeration. We searched electronically MEDLINE, EMBASE and the Cochrane Controlled Trials Registers from 1996 to July 2019 for potentially relevant studies using a focused search strategy, whose details are provided in the online supplement. Bibliography of the selected studies was inspected for potential inclusion of other trials.

### Study selection, quality assessment and data extraction

A primary search was conducted by two investigators (LB and PP) who evaluated the adherence to the inclusion criteria solving discrepancies by consensus, and when consensus was not reached a third investigator was consulted (PRMR). The trials were also assessed for potential sources of bias using the Cochrane Collaboration risk of bias instrument, assessing random sequence generation and allocation concealment, blinding of participants, personnel and outcome assessors, presence of incomplete outcome data or selective reporting and other potential sources of bias. Data extraction was performed independently by two authors (VT and MM), and discrepancies were solved by consensus.

### Outcomes

The primary outcome was mortality at 28 days, substituted when not reported by mortality at 30 days, intensive care unit (ICU) discharge, hospital discharge or at 60 days, in this sequence as available in the analysed trial. This collapsed mortality end point was recently proposed by a panel of experts of the ‘Mechanical Ventilation for ARDS Clinical Practice Guideline’ taskforce [10]. Secondary outcomes were incidence of barotrauma, extrapulmonary complications and gas exchange and ventilation parameters. Barotrauma was defined as pneumothorax, pneumomediastinum or subcutaneous emphysema. We recorded the different definitions of extrapulmonary complications in the different included studies; however, as detailed in the results section, their heterogeneity was too high to perform a formal meta-analysis. We collected patients’ characteristics at baseline, and ventilation and blood gas analysis data at 1, 3 and 7 days, or at the closest reported time point.

### Subgroup analyses

We stratified the studies according to the type of intervention (*higher* PEEP, RMs or their combination). We further performed a pre-planned stratification only including patients with PaO_2_/FIO_2_ ≤ 200 mmHg at randomisation and a post-hoc stratification comparing studies that titrated *higher PEEP* based on oxygenation or respiratory mechanics goals. Outcome data for subgroups were collected where available. For three trials [11–13], data of this sub-group was extracted from the pooled stratum reported in an individual patient meta-analysis [9].

### Sensitivity analyses

To assess whether the control groups were representative of the current practice of ventilation of ARDS patients, we compared their baseline characteristics and ventilator settings after enrolment with the median values extrapolated from the *‘Large observational study to UNderstand the Global impact of Severe Acute respiratory FailurE’* (LUNG SAFE) [1]. Moreover, we performed a meta-regression to evaluate the influence on the effect size of the following parameters: method of setting PEEP, fraction of pulmonary ARDS at enrolment, use of recruitment manoeuvres, PaO_2_/FIO_2_ ratio at randomisation, PEEP difference between treatment and control at the time point closest to day 1.

Data synthesis and analysis

For dichotomous outcomes, we computed the relative risks (RRs) with their 95% confidence intervals (CIs). For continuous variables describing patients’ characteristics and parameters at baseline and at different time points, we computed the pooled average and standard deviation (SD) of each group and their mean difference. All estimates were calculated with a mixed-effects model using the DerSimonian-Laird method and a continuity correction constant of 0.5. Potential bias for the primary outcome was examined with a funnel plot of treatment effect versus study precision, with an Egger test for plot asymmetry. Subgroups were compared with the Cochran’s *Q* test, and residual heterogeneity was assessed with the *I*^*2*^ statistics and *Q* test. Comparisons between the control group and the median values reported in the LUNG SAFE study were performed with one-sampled Student’s *t* tests. We conducted a formal trial sequential analysis (TSA) limiting the global type I error to 5%, computing the two-sided *α*-spending boundaries and futility area with the O’Brien-Fleming function. This method provides conservative CI estimates for the effect size, similar to what is done in ad interim analyses in RCTs. We hypothesized a pooled mortality rate of 35% in the control arm, and we aimed to achieve 90% power (1-β) to detect a 25% relative risk reduction in the intervention arm.

All analyses were performed with R 3.2.3 and the metafor and meta packages (The R Foundation for Statistical Computing, www.r-project.org), RevMan 5.3 (Cochrane Collaboration, Copenhagen, Denmark) and TSA 0.9.5.10 (Copenhagen Trial Unit, Copenhagen, Denmark). Statistical significance was considered for two–tailed *p* < 0.05. The protocol had been registered in the Prospero database (CRD42017082035).

## Results

**Fig. 1:**
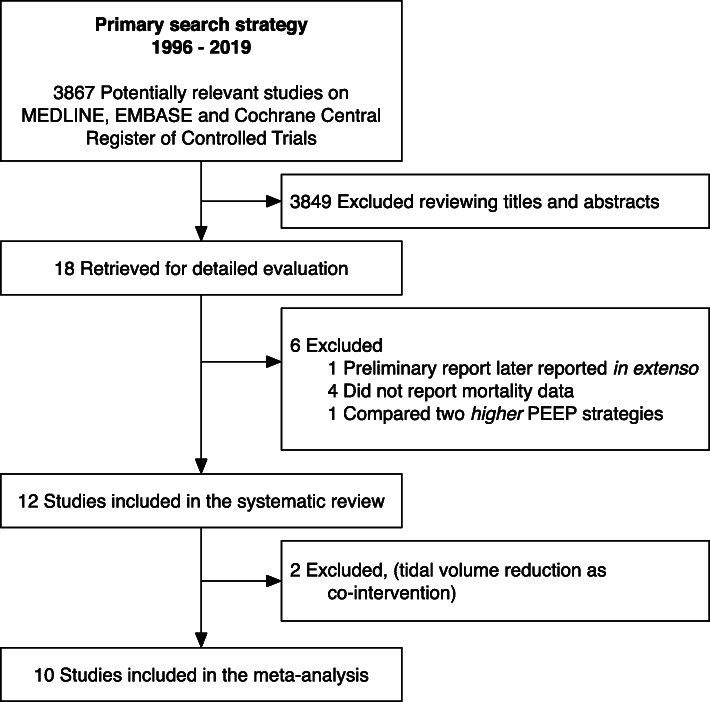
Study inclusion flowchart

Figure 1 depicts the study inclusion flow, and Table 1 shows the description of the included studies. Overall, risk of bias was moderate-low (eFigures 1 and 2). We found six studies using *higher* PEEP plus RMs [11, 14–18], three using *higher* PEEP alone [12, 13, 19] and one using RMs alone [20]. We also found two studies [4, 5] in which *higher* PEEP and RMs were used in conjunction with *V*_T_ reduction, but we did not consider these studies in the meta-analysis since their inclusion resulted in high clinical and statistical heterogeneity (see eFigure 3).

**Table 1:** Study description

				Treatments				
				Intervention group			Control group	
Study	PaO_2_/FIO_2_ (mmHg)	Patients (centres)	Mortality time points	Aim	Ventilation strategy	Recruitment manoeuvres	Aim	Ventilation strategy
Studies investigating higher PEEP with TV reduction								
Amato 1998*	< 200	53 (2)	28 days In hospital In ICU	Maintaining lung recruitment with higher PEEP, lower TV plus RMs	TV < 6 mL/kg PEEP = P_FLEX_ + 2 cmH_2_O P_DRIV_ < 20 cmH_2_O P_PEAK_ < 40 cmH_2_O	CPAP of 35–40 cmH_2_O for 40s	Maintaining oxygenation, using low PEEP and high TV	TV = 12 mL/kg PEEP ≥ 5 cmH_2_O Stepwise PEEP titration table targeting PaO_2_ ≥ 80 mmHg
Villar 2006*	< 200	95 (8)	In-hospital In ICU	Maintaining oxygenation while increasing lung recruitment, with higher PEEP and lower TV	TV 5–8 mL/kg PEEP = P_FLEX_ + 2 cmH_2_O	No	Maintaining oxygenation using low PEEP and high TV	TV 9–11 mL/kg PEEP ≥ 5 cmH_2_O
Studies investigating higher PEEP without recruitment manoeuvres								
Brower 2004	< 300	549 (23)	In-hospital	Maintaining oxygenation prioritizing PEEP over FIO_2_ (higher PEEP levels)	TV 6 mL/kg Higher PEEP/FIO_2_ table	Only in first 80 patients	Maintaining oxygenation prioritizing FIO_2_ over PEEP (lower PEEP levels)	TV 6 mL/kg Lower PEEP/FIO_2_ table P_PLAT_ < 30 cmH_2_O
Mercat 2008	< 300	767 (37)	28 days in-hospital	Increasing alveolar recruitment while limiting hyperinflation with higher PEEP	TV 6 mL/kg Highest PEEP resulting in P_PLAT_ < 28––30 cmH_2_O	Not recommended	Minimizing alveolar distension with a moderate-low PEEP strategy	TV 6 mL/kg PEEP 5–9 cmH_2_O
Talmor 2008	< 300	61 (1)	28 days	Maintaining oxygenation setting PEEP based on transpulmonary pressure	TV 6 mL/kg PEEP set to achieve: End-expiratory transpulmonary pressure 0–10 cmH_2_O End-inspiratory transpulmonary pressure < 25 cmH_2_O	No	Maintaining oxygenation	TV 6 mL/kg Lower PEEP/FIO_2_ table P_PLAT_ < 30 cmH_2_O
Studies investigating higher PEEP with recruitment manoeuvres								
Meade 2008	< 250	983 (30)	28 days In-hospital In ICU	Maintaining an ‘open-lung approach’ based on oxygenation goals	TV 6 mL/kg Higher PEEP/FIO_2_ table P_PLAT_ < 40 cmH_2_O	CPAP 40 cmH_2_O for 40 s	Maintaining oxygenation	TV 6 mL/kg Lower PEEP/FIO_2_ table P_PLAT_ < 30 cmH_2_O
Huh 2009	< 200	57 (1)	28 days 60 days In ICU	Individualisation of PEEP according to compliance and oxygenation	TV 6 mL/kg PEEP set with a decremental trial at the lowest value without decrease in saturation or compliance	PEEP increased from baseline to 25 cmH_2_O	Maintaining oxygenation	TV 6 mL/kg Lower PEEP/FIO_2_ table
Hodgson 2011	< 200	20 (1)	In-hospital	Lung recruitment and individualisation of PEEP according to oxygenation	TV < 6 mL/kg Decremental PEEP until desaturation ≥1%	PEEP increased to 40 cmH_2_O and reduced to 15 cmH_2_O	Maintaining oxygenation	TV 6 mL/kg Lower PEEP/FIO_2_ table
Kacmarek 2016	< 200	200 (20)	28 days 60 days In-hospital In ICU	Maintaining an ‘open-lung approach’	TV 6 mL/kg Decremental PEEP to the best dynamic compliance	P_PEAK_ 50-60 cmH_2_O PEEP 35–45 cmH_2_O	Maintaining oxygenation	TV 6 mL/kg Lower PEEP/FIO_2_ table
Cavalcanti 2017	< 200	1004 (120)	28 days In-hospital In ICU	Maintaining an ‘open-lung approach’	TV 6 mL/kg PEEP ≥ 11 cmH_2_O, set to the lowest P_DRIV_ in a decremental titration	P_PLAT_ ≤ 50 cmH_2_O PEEP increased to 35 cmH_2_O	Maintaining oxygenation	TV 6 mL/kg Lower PEEP/FIO_2_ table
Hodgson 2019	< 200		28 days 60 days In-hospital In ICU 6 months	Lung recruitment and individualisation of PEEP according to oxygenation	TV < 6 mL/kg Decremental PEEP until desaturation ≥ 2%	PEEP increased to 40 cmH_2_O and reduced to 15 cmH_2_O	Maintaining oxygenation	TV 6 mL/kg Lower PEEP/FIO_2_ table
Studies investigating recruitment manoeuvres alone								
Xi 2010	200	110 (14)	28 days In-hospital In ICU	Maintaining lung recruitment with RMs only	TV 6–8 mL/kg Lower PEEP/FIO_2_ table RM performed by CPAP	CPAP of 40 cmH_2_O for 40s	Maintaining oxygenation	TV 6-8 mL/kg Lower PEEP/FIO_2_ table P_PLAT_ < 30 cmH_2_O

*Excluded from the meta-analysis as the intervention group received a lower tidal volume compared to the control group

*PEEP* positive end-expiratory pressure, *TV* tidal volume, *CPAP* continuous positive airway pressure, *P*_*FLEX*_ lower inflection point, *P*_*DRIV*_ driving pressure, *P*_*PEAK*:_peak pressure, *RM*, recruitment manoeuvre.

**Fig. 2:**
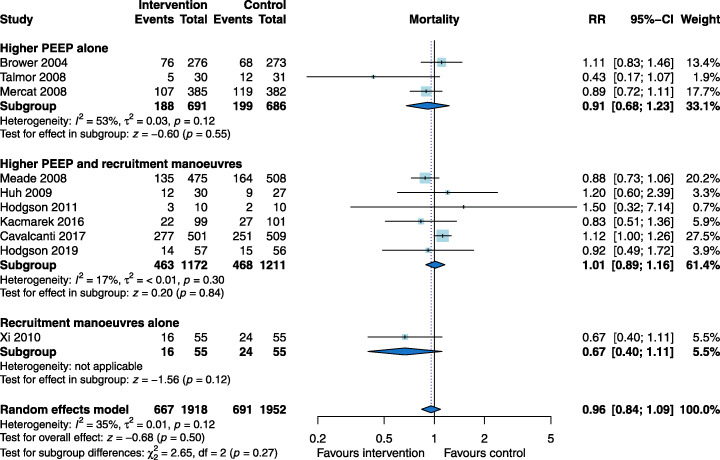
Forest plot for mortality (collapsed at 28 days, ICU discharge, hospital discharge or 60–days). Studies are stratified according to whether higher PEEP and recruitment manoeuvres were used separately or as a bundle of interventions. ICU, intensive care unit; PEEP, positive end-expiratory pressure

**Fig. 3:**
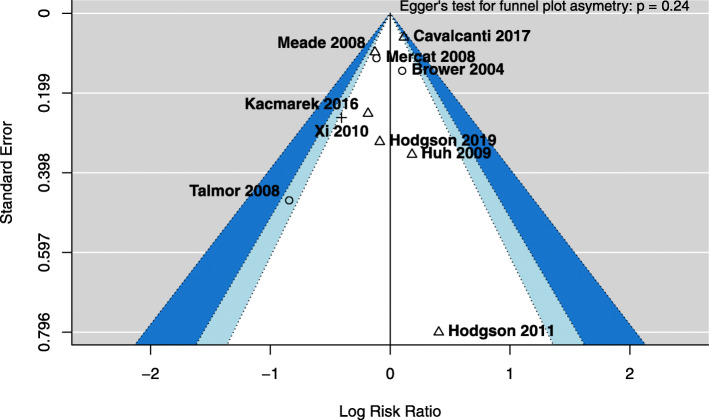
Funnel plot for mortality (collapsed at 28 days, ICU discharge, hospital discharge or 60 days). Shapes represent different interventions: higher PEEP alone (circle), recruitment manoeuvres alone (cross) or both (triangle). Dotted lines represent the 90%, 95% and 99% pseudo confidence interval regions. ICU, intensive care unit; PEEP, positive end-expiratory pressure

We included 3870 patients in the meta-analysis, whose baseline characteristics are reported in Table 2. As shown in Table 3, in the intervention *versus* control group at days 1, 3 and 7 from randomisation, there were no differences in *V*_T_ size or respiratory rate, while PEEP and PaO_2_/FIO_2_ ratio were consistently higher. Driving pressure was lower in the intervention group at days 1 and 3, but not at day 7. Plateau pressure was higher in the intervention group at days 1 and 3, but not at day 7. We did not observe differences in mortality (RR 0.96, 95% confidence interval, CI [0.84–1.09], *p* = 0.50, Fig. 2, funnel plot in Fig. 3) nor in incidence of barotrauma (RR 1.22, 95% CI [0.93–1.61], *p* = 0.16, eFigure 4) in the pooled analysis. Stratification according to the different combination of PEEP/RM intervention reduced statistical heterogeneity, but still no differences in mortality (Fig. 2) nor barotrauma (eFigure 4) were observed. Mortality at day 28 (eFigure 5), ICU discharge (eFigure 6), hospital discharge (eFigure 7) and at day 60 (eFigure 8) was not different between groups. No differences in mortality or incidence of barotrauma were observed when analysis was restricted to studies including only patients with PaO_2_/FIO_2_ below 200 mmHg at enrolment (e-Figures 9 and 10). Extrapulmonary complications, ventilator- and organ failure-free days were reported heterogeneously from a clinical and statistical point of view, and a formal meta-analysis was not feasible.

**Table 2:** Baseline characteristics of patients included in the meta-analysis

Parameter	Intervention (*n* = 1918)	Control (*n* = 1952)
Age, years	55.7 (3.2) [*n* = 1919]	56.1 (5.3) [*n* = 1952]
Women, No (%)	732 (38) [*n* = 1918]	750 (38) [*n* = 1952]
PaO_2_/FIO_2_ at enrolment, mmHg	135.8 (14.4) [*n* = 1859]	134.7 (17.4) [*n* = 1892]
Respiratory system compliance, mL/cmH_2_O	32.9 (5.1) [*n* = 969]	32.0 (4.4) [*n* = 975]
Causes of lung injury		
Pneumonia, No. (%)	920 (49) [*n* = 1878]	933 (49) [*n* = 1911]
Aspiration, No. (%)	175 (13) [*n* = 1373]	185 (13) [*n* = 1376]
Sepsis, No. (%)	495 (26) [*n* = 1908]	538 (28) [*n* = 1942]
Multiple trauma, No. (%)	54 (4) [*n* = 1394]	72 (5) [*n* = 1432]
Ventilation parameters		
Tidal volume, mL/kg of predicted body weight	7.3 (1.1) [*n* = 1909]	7.4 (1.2) [*n* = 1942]
Set PEEP, cmH_2_O	11.2 (1.8) [*n* = 1643]	11.1 (2.1) [*n* = 1679]
Driving pressure, cmH_2_O	15.4 (2.4) [*n* = 1575]	15.2 (2.1) [*n* = 1615]
Plateau pressure, cmH_2_O	27.0 (2.8) [*n* = 1575]	26.8 (2.7) [*n* = 1615]

Data are mean (standard deviation) or frequency (proportion). Number of patients for each variable is also reported, as data was missing or not reported as mean in all studies. Values are estimated means (standard deviations) calculated with a mixed-effects model using the DerSimonian-Laird method. *PEEP* positive end-expiratory pressure

**Table 3:** Ventilator and blood gas analysis parameters collected at day 1, 3 and 7

	Day 1			Day 3			Day 7		
Variable	Intervention	Control	*p*	Intervention	Control	*p*	Intervention	Control	*p*
Tidal volume, mL/kg of predicted body weight	6.1 (0.3) [*n* = 1762]	6.2 (0.3) [*n* = 1778]	0.07	6.3 (0.4) [*n* = 1475]	6.3 (0.3) [*n* = 1553]	0.62	6.6 (0.4) [*n* = 926]	6.5 (0.3) [*n* = 1028]	0.93
Set PEEP, cmH_2_O	14.8 (1.2) [*n* = 1813]	10.1 (2.1) [*n* = 1839]	< 0.001	13.2 (1.4) [*n* = 1627]	9.4 (1.8) [*n* = 1663]	< 0.001	10.4 (1.4) [*n* = 1142]	8.6 (1.7) [*n* = 1213]	< 0.001
Driving pressure, cmH_2_O	12.6 (1.1) [*n* = 1793]	14.0 (0.9) [*n* = 1826]	< 0.001	12.7 (2.2) [*n* = 1599]	14.1 (1.3) [*n* = 1640]	0.013	13.8 (2.8) [*n* = 1114]	14.7 (1.8) [*n* = 1193]	0.26
Plateau pressure, cmH_2_O	27.6 (1.1) [*n* = 1740]	24.4 (1.9) [*n* = 1715]	<0.001	26.1 (1.6) [*n* = 1445]	23.7 (1.8) [*n* = 1511]	0.001	24.4 (2.5) [*n* = 873]	23.6 (1.9) [*n* = 965]	0.176
Respiratory rate, min^-1^	27.4 (2.2) [*n* = 1800]	27.3 (1.7) [*n* = 1841]	0.57	26.4 (2.2) [*n* = 1606]	27.3 (1.3) [*n* = 1680]	0.50	26.0 (1.7) [*n* = 1136]	26.3 (0.8) [*n* = 1193]	> 0.99
FIO_2_	0.50 (0.05) [*n* = 1180]	0.59 (0.05) [*n* = 1193]	< 0.001	0.45 (0.04) [*n* = 1065]	0.53 (0.04) [*n* = 1088]	< 0.001	0.44 (0.05) [*n* = 739]	0.50 (0.04) [*n* = 785]	0.023
PaO_2_/FIO_2_, mmHg	208.3 (16.6) [*n* = 1719]	152.6 (10.9) [*n* = 1649]	< 0.001	224.1 (24.0) [*n* = 1537]	168.0 (12.5) [*n* = 1575]	< 0.001	216.1 (23.5) [*n* = 1101]	185.0 (17.1) [*n* = 1179]	< 0.001
PaO_2_, mmHg	95.1 (9.6) [*n* = 1180]	82.1 (3.9) [*n* = 1200]	< 0.001	91.3 (12.0) [*n* = 1066]	87.4 (10.2) [*n* = 1089]	0.54	81.8 (7.0) [*n* = 726]	82.0 (6.0) [*n* = 769]	0.80
PaCO_2_, mmHg	47.1 (4.9) [*n* = 1785]	45.6 (3.5) [*n* = 1809]	0.07	45.3 (3.6) [*n* = 1574]	46.0 (3.2) [*n* = 1611]	0.37	44.6 (3.2) [*n* = 1168]	46.1 (3.1) [*n* = 1244]	0.013
pHa	7.34 (0.04) [*n* = 1785]	7.35 (0.03) [*n* = 1809]	0.033	7.38 (0.02) [*n* = 1575]	7.38 (0.03) [*n* = 1612]	0.99	7.40 (0.01) [*n* = 1168]	7.40 (0.02) [*n* = 1244]	0.53

Values are estimated means (standard deviations) calculated with a mixed-effects model using the DerSimonian-Laird method. Number of patients at each time-point is also reported, as in some study or time point values were missing or not reported as means (standard deviations). *PEEP* positive end-expiratory pressure

**Fig. 4:**
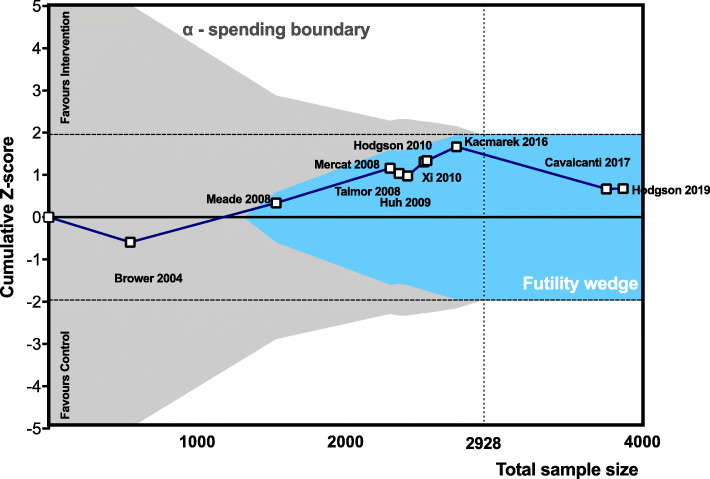
Trial sequential analysis assessing the effects of PEEP and/or recruitment manoeuvres on mortality (collapsed at 28 days, ICU discharge, hospital discharge). Required information size of 2487 patients (dotted line) is calculated for a relative risk reduction of 25%, *α* = 5%, power (1-β) = 80%. The Z-line (blue line) of the cumulative meta-analysis of 3757 patients did not cross the efficacy monitoring boundaries for benefit or harm (grey area) but entered the futility wedge (light blue area). Horizontal dashed lines represent the conventional level of significance (*p* = 0.05)

In the trial sequential analysis, the required information size of 2928 was reached, and the cumulative Z-score did not cross the alpha-spending nor the conventional 95% boundaries, meaning that significance was not reached, but entered the futility wedge (Fig. 4).

### Sensitivity and post-hoc analyses

At the meta-regression analysis, no association with mortality nor barotrauma was found for PEEP titration method, percent of patients with pulmonary *versus* extra-pulmonary ARDS, use of RMs, PaO_2_/FIO_2_ ratio at randomisation and difference in PEEP at day 1 (*p* > 0.29 for all covariates, details in e-tables 2 and 3).

No differences in mortality were observed when stratifying the analysis according to whether PEEP was set based on oxygenation or respiratory mechanics targets (e-Figures 11 and 12).

Compared to the population of the LUNG SAFE study, the control group of this meta-analysis included patients ventilated with lower *V*_T_ (*p* < 0.001), while no differences were observed in age (*p* = 0.36), PEEP levels (*p* = 0.42), plateau pressure (*p* = 0.53), respiratory rate (*p* = 0.68), FIO_2_ (*p* = 0.23), PaO_2_/FIO_2_ ratio (*p* = 0.44) and PaCO_2_ (*p* = 0.91).

## Discussion

The main finding of the present meta-analysis was that, in unselected patients with ARDS who were mechanically ventilated with protective low *V*_T_, the use of *higher* PEEP and/or RMs does not result in mortality reduction nor incidence of barotrauma compared to a strategy using a PEEP level aimed at achieving minimal acceptable oxygenation goals.

This meta-analysis has several strengths. First, we restricted it to trials not changing *V*_T_ between groups, to avoid a relevant confounding factor. Second, we stratified the studies according to the type of co-interventions when feasible, to reduce the clinical and statistical heterogeneity. Third, we conducted a formal trial sequential analysis to assess the conclusiveness of the available evidence. Fourth, we conducted several pre-planned and post-hoc analyses including meta-regression and conventional stratification to explore for meaningful associations.

In the pooled analysis, the average PEEP was around 15 and 10 cmH_2_O in the intervention and control groups, respectively. The latter reflects the current practice of ventilation in ARDS patients, while *V*_T_ was lower than what is currently used [1]. As previously discussed in another systematic review, mortality improvement was only observed when PEEP increase is used in conjunction with a reduction of *V*_T_ size [21]. Regardless of the combination of PEEP/RM interventions, we did not observe any improvement in mortality when pooling data from different studies. In an individual patient meta-analysis published in 2010 [9] including patients enrolled in three trials [11–13], an improvement in mortality was observed in patients with a PaO_2_/FIO_2_ ≤ 200 mmHg at randomisation. However, following these encouraging findings, all studies published thereafter only included patients with moderate to severe ARDS, without showing improvements in mortality [15–17, 20]. Nonetheless, a recent guideline recommends the use of *higher* PEEP levels in moderate to severe ARDS patients [8], but stresses the importance to balance between the advantages in lung recruitment and the risk of reaching elevated plateau pressures. In our pooled analysis, average plateau pressure was below 30 cmH_2_O in both arms; however one trial reports that, with *higher* PEEP, plateau pressure can transiently cross this threshold more frequently as compared to lower PEEP [17].

A recent study found that high driving pressure (plateau pressure minus PEEP) is strongly associated with ARDS mortality [22]. Therefore, it has been proposed that strategies aimed at reducing driving pressure could improve mortality, but this is matter of debate [23]. In our analysis, we observed that patients in the intervention group received significantly higher PEEP at all the analysed time points until day 7, although the magnitude of such difference decreased over time. This resulted in a reduction of driving pressure of as little as 1 cmH_2_O at days 1 and 3, and this difference was no longer significant at day 7. Driving pressure was proposed as a surrogate of dynamic strain; thus, its reduction through lung recruitment, achieved with *higher* PEEP or RMs, could be lung protective. Nevertheless, few studies described the magnitude of PEEP change resulting from the PEEP titration method, and the effect of PEEP could have been confounded by the fact that some patient received the treatment, according to the intervention arm protocol, also in case of a limited or absent response to PEEP (i.e. driving pressure reduction or oxygenation improvement). Thus, it is possible that the price paid in terms of exposure to higher static strain and barotrauma during RMs offsets the benefits of obtaining an *‘*open lung*’* [24]. Moreover, it has been recently observed that in ARDS patients admitted to the intensive care unit, differently from experimental models where PEEP is set immediately after the induction of lung injury, part of the lung collapse cannot be reverted after reaching 40 cmH_2_O airway pressure, thus questioning the possibility of achieving an *‘*open lung’ [25]. In a single study, PEEP was titrated based on the oesophageal pressure, and this resulted in a much wider distribution of PEEP levels [19]. However, when this strategy was compared to a higher PEEP/FIO_2_ table in a larger cohort, no differences in mortality were observed [26]. We opted not to include the latter trial in the present meta-analysis because it did not fulfil the inclusion criteria, as the control group received *higher* PEEP, PEEP levels in the intervention and control groups were similar and the aim was to individualise rather than indiscriminately increasing the PEEP level.

In the meta-regression, the PEEP difference between intervention and control group at day 1 was not associated with increased risk of barotrauma. Moreover, in the study in which the incidence of barotrauma was the highest [17], PEEP difference was as low as 3 cmH_2_O immediately after randomisation. Some authors proposed that the increased incidence of barotrauma and the higher mortality observed in the intervention group of such study could be explained by alveolar recruitment manoeuvres, not by the PEEP difference [27]. In that trial, the RMs were performed with an abrupt increase of PEEP to 35 cmH_2_O [17]. In this line, an abrupt increase of PEEP is associated with lung inflammation in experimental ARDS [28] and increased postoperative pulmonary complications in obese patients [29]. Three previous high-quality meta-analyses concluded that RMs could decrease mortality in ARDS patients, although evidence is inconclusive and of low quality [10, 30, 31]. However, none of these meta-analyses included the most recent trials [17, 18], and in only single study RMs were used without other co-interventions, with no effects on 28-days or in-hospital mortality [20]. Moreover, as detailed in Table 1, different techniques of recruitment manoeuvres were used in the 6 studies comprising them in the intervention arm. We cannot exclude that the type of recruitment manoeuvre can influence the clinical outcome and the level of PEEP identified as ‘best PEEP’.

The trial sequential analysis showed that the optimum sample size was reached, though the high heterogeneity of techniques for setting PEEP and performing RMs across studies suggests caution before considering evidence as definitive. The generalisability of the findings of this meta-analysis could be limited by some factors: (1) we were unable to analyse patient data individually; thus, we could have missed specific sub-groups of patients in which *higher* PEEP and/or RMs are beneficial; (2) in most trials, ARDS criteria were only assessed at inclusion; thus, its incidence could have been over-estimated; (3) in several studies, very severe patients were excluded; (4) several secondary outcomes could not be assessed systematically and (5) the type of recruitment manoeuvre differed across trials.

## Conclusions

The current evidence does not support the routine use of higher PEEP levels and recruitment manoeuvres in unselected patients with ARDS who are mechanically ventilated with protective low tidal volume.

## Supplementary information

**Additional file 1.** Additional analyses.

## Data Availability

Availability of data and materials does not apply.

## Abbreviations

ARDS: Acute respiratory distress syndrome
ICU: Intensive care unit
PEEP: Positive end-expiratory pressure
RCT: Randomized controlled trial
RM: Recruitment manoeuvre
TSA: Trial sequential analysis
