# Supplementary material for: Effects of higher PEEP and recruitment manoeuvres on mortality in patients with ARDS: a systematic review, meta-analysis, meta-regression and trial sequential analysis of randomized controlled trials
Source: Intensive Care Med Exp. 2020 Dec 18;8(Suppl 1):39. doi: 10.1186/s40635-020-00322-2 (PMC7746429; doi:10.1186/s40635-020-00322-2)
Supplement: Supplementary file 1 — Additional file 1. Additional analyses. [file 40635_2020_322_MOESM1_ESM.docx]

Effects of PEEP and Recruitment Manoeuvres on Mortality in ARDS Patients:

A Meta-Analysis, Meta-Regression and Trial Sequential Analysis of Randomized Controlled Trials

Lorenzo Ball^1,2,3^, Ary Serpa Neto^3^, Valeria Trifiletti^1^, Maura Mandelli^1^, Iacopo Firpo^1^, Chiara Robba^2^, Marcelo Gama de Abreu^5^, Marcus J. Schultz^3,6^, Nicolò Patroniti^1,2^, Patricia R.M. Rocco^7^, Paolo Pelosi^1,2^

For the PROVE Network*

**University of Genova, Genova, Italy**

^1^Department of Surgical Sciences and Integrated Diagnostics

**Ospedale Policlinico San Martino IRCCS per l’Oncologia e le Neuroscienze, Genova, Italy**

^2^Anesthesia and Intensive Care

**Academic Medical Center, University of Amsterdam, Amsterdam, Netherlands**

^3^Department of Intensive Care

**Hospital Israelita Albert Einstein, São Paulo, Brazil**

^4^Department of Critical Care Medicine

**University Hospital Carl Gustav Carus, Technische Universität Dresden, Dresden, Germany**

^5^Pulmonary Engineering Group, Department of Anaesthesiology and Intensive Care Medicine

**Mahidol University, Bangkok, Thailand**

^6^Mahidol Oxford Tropical Medicine Research Unit (MORU)

**Carlos Chagas Filho Institute of Biophysics, Federal University of Rio de Janeiro, Rio de Janeiro, Brazil**

^7^Laboratory of Pulmonary Investigation

Table Contents

e-Table 1 - Patients, interventions, comparisons and outcome (PICO) 4

Search strategy 4

Risk of bias assessment 5

e-Figure 1 - Risk of bias graph 5

e-Figure 2 - Risk of bias summary 6

e-Figure 3 - Forrest plot for mortality, including studies with tidal volume reduction as co-intervention 7

e-Figure 4 - Forest plot for barotrauma 8

Forest plot for mortality at different time-points 9

e-Figure 5 - Forest plot for mortality at day 28 9

e-Figure 6 - Forest plot for mortality at ICU discharge 10

e-Figure 7 - Forest plot for mortality at hospital discharge 11

e-Figure 8 - Forest plot for mortality at day 60 12

Studies including patients with PaO_2_/FIO_2_ < 200 mmHg 13

e-Figure 9 - Forest plot mortality, studies with PaO_2_/FIO_2_ below 200 mmHg 13

e-Figure 10 - Forest plot for barotrauma, studies with PaO_2_/FIO_2_ below 200 mmHg 14

Meta-regression analysis 15

e-Table 2 - Meta-regression for mortality 15

e-Table 3 - Meta-regression for barotrauma 15

Studies titrating PEEP based on oxygenation vs. mechanics 16

e-Figure 13 - Forest plot for mortality, studies titrating PEEP based on oxygenation vs. mechanics 16

e-Figure 14 - Forest plot for barotrauma, studies titrating PEEP based on oxygenation vs. mechanics 17

# e-Table 1 - Patients, interventions, comparisons and outcome (PICO)

| **Questions** | **Description** |
| --- | --- |
| Population | Mechanically ventilated, adults meeting the definition of acute respiratory distress syndrome. |
| Interventions | Higher Positive End-expiratory Pressure (PEEP), defined as any strategy resulting in, or aimed at, obtaining PEEP levels higher than those achieved in the control group.  and / or  Recruitment Manoeuvres, namely any transient increase in airway pressure aimed at restoring or improving lung aeration. |
| Comparator | Lower PEEP, titrated to oxygenation goals, no recruitment manoeuvers |
| Outcomes | Mortality  Barotrauma  Oxygenation (PaO_2_/FiO_2_) |

**e-Table 1**: Description of patient, interventions, comparisons and outcome measurements.

# Search strategy

We searched MEDLINE, EMBASE and the Cochrane Central Register of Controlled Trials with the following keywords, restricting from 1996 to June 2019: ((((positive end-expiratory pressure* OR PEEP) OR (open lung strateg* OR open lung approach) OR (recruitment AND (maneuver* OR manoeuvre*))) AND (ARDS OR acute respiratory distress syndrome OR ALI OR acute lung injury

# Risk of bias assessment

## e-Figure 1 - Risk of bias graph

##

**e-Figure 1**: Risk of bias graph: review authors’ judgements about each risk of bias item presented as percentages across all included studies.

## e-Figure 2 - Risk of bias summary

**e-Figure 2:** Risk of bias summary: review authors’ judgements about each risk of bias item for each included study.

# e-Figure 3 - Forrest plot for mortality, including studies with tidal volume reduction as co-intervention

**e-Figure 3:** Forest plot for mortality (collapsed at 28 – days, ICU discharge, hospital discharge or 60 – days). Studies are stratified according to whether VT was changed or not in the intervention vs. control group.

# e-Figure 4 - Forest plot for barotrauma

**e-Figure 4:** Forest plot for barotrauma. Studies are stratified according to whether higher PEEP and recruitment manoeuvres were used separately or as a bundle of interventions.

# Forest plot for mortality at different time-points

## e-Figure 5 - Forest plot for mortality at day 28

**e-Figure 5:** Forest plot for mortality at 28 days.

## e-Figure 6 - Forest plot for mortality at ICU discharge

**e-Figure 6:** Forest plot for mortality at ICU discharge.

## e-Figure 7 - Forest plot for mortality at hospital discharge

**e-Figure 7:** Forest plot for mortality at hospital discharge.

## e-Figure 8 - Forest plot for mortality at day 60

**e-Figure 8:** Forest plot for mortality at 60 days.

# Studies including patients with PaO_2_/FIO_2_ < 200 mmHg

## e-Figure 9 - Forest plot mortality, studies with PaO_2_/FIO_2_ below 200 mmHg

**e-Figure 9:** Forest plot for mortality (collapsed at 28 – days, ICU discharge, hospital discharge or 60 – days). Studies included only with PaO_2_/FIO_2_ < 200 mmHg.

## e-Figure 10 - Forest plot for barotrauma, studies with PaO_2_/FIO_2_ below 200 mmHg

**e-Figure 10:** Forest plot for barotrauma. Studies included only with PaO_2_/FIO_2_ < 200 mmHg.

# Meta-regression analysis

## e-Table 2 - Meta-regression for mortality

| **Variable** | **β [95% confidence interval]** | **p** |
| --- | --- | --- |
| PEEP set on mechanics (vs. oxygenation) | 0.12 [-0.52 - 0.77] | 0.71 |
| Prevalence of pulmonary ARDS (%) | 0.01 [-0.01 - 0.02] | 0.57 |
| Use of RM (vs no RM) | 0.01 [-1 - 1.03] | 0.98 |
| PaO_2_/FIO_2_ ratio at randomisation (per mmHg) | 0.02 [-0.02 - 0.05] | 0.42 |
| PEEP difference at day 1 (per cmH_2_O) | -0.11 [-0.39 - 0.18] | 0.46 |

**e-Table 2**: Meta-regression for mortality (collapsed at 28 – days, ICU discharge, hospital discharge or 60 – days). PEEP: positive end-expiratory pressure; RM: recruitment manoeuvres.

## e-Table 3 - Meta-regression for barotrauma

| **Variable** | **β [95% confidence interval]** | **p** |
| --- | --- | --- |
| PEEP set on mechanics (vs. oxygenation) | 0.39 [-1.27 - 2.04] | 0.65 |
| Prevalence of pulmonary ARDS (%) | -0.02 [-0.07 - 0.02] | 0.29 |
| Use of RM (vs no RM) | 0.82 [-1.47 - 3.1] | 0.49 |
| PaO_2_/FIO_2_ ratio at randomisation (per mmHg) | -0.03 [-0.12 - 0.07] | 0.59 |
| PEEP difference at day 1 (per cmH_2_O) | 0.3 [-0.34 - 0.95] | 0.35 |

**e-Table 3**: Meta-regression for barotrauma. PEEP: positive end-expiratory pressure; RM: recruitment manoeuvres.

# Studies titrating PEEP based on oxygenation vs. mechanics

## e-Figure 11 - Forest plot for mortality, studies titrating PEEP based on oxygenation vs. mechanics

**e-Figure 11**: Forest plot for mortality (collapsed at 28 – days, ICU discharge, hospital discharge or 60 – days). Studies are stratified according to whether PEEP titrated based on oxygenation goals or respiratory mechanics.

## e-Figure 12 - Forest plot for barotrauma, studies titrating PEEP based on oxygenation vs. mechanics

**e-Figure 12**: Forest plot for barotrauma. Studies are stratified according to whether PEEP titrated based on oxygenation goals or respiratory mechanics.
